# Cause rare d'occlusion mécanique chez l'enfant: à propos d'un cas

**DOI:** 10.11604/pamj.2021.38.71.27634

**Published:** 2021-01-21

**Authors:** Amina Mouffak, Siham Salam, Amine Fahl, Kamilia Chbani, Dalal Laoudiyi, Lahcen Ouzidane

**Affiliations:** 1 Service de la Radiologie Pédiatrique, Hôpital d'Enfants Abderrahim Harouchi, Casablanca, Maroc

**Keywords:** Bézoard, syndrome de Rapunzel, occlusion, *case report*, Bezoar, Rapunzel syndrome, occlusion, case report

## Abstract

Les bézoards correspondent à la concrétion de substances ingérées non digestibles dans le tractus gastro-intestinal. Le trichobézoard est la forme la plus fréquente des bézoards, et correspond à l'ingestion de cheveux, poils ou fibres de tapis ou moquette. Principalement de localisation intra-gastrique, il existe des formes rares d'extension duodénale ou à l'intestin grêle définies par le syndrome de Rapunzel. L'imagerie notamment scanographique est typique et joue un double rôle diagnostique et pronostique. Nous rapportons le cas d'une enfant âgée de 13 ans hospitalisée pour un syndrome occlusif sur un trichobézoard.

## Abstract (en)

Gastrointestinal (GI) bezoars are aggregates of undigested material found in the GI tract. Trichobezoar is the most common type of bezoars and consists of ingested hair, carpet fibers or fitted carpet fibers. They are mainly located in the gastric region, rare forms extend to the duodenum or small intestine and are described as Rapunzel syndrome. Typical CT imaging features play a diagnostic and prognostic role. We report the case of a 13-year-old girl hospitalized for occlusive syndrome due to trichobezoar.

## Introduction

Le trichobézoard correspond à l'ingestion de cheveux, poils ou fibres de tapis ou moquette et représente la forme la plus fréquente des bézoards. La symptomatologie clinique est non spécifique et variée en fonction du site de localisation qui reste principalement intra-gastrique avec possibilité d'autres localisations tout le long du tractus digestif. Nous rapportons le cas d'une enfant âgée de 13 ans hospitalisée pour un syndrome occlusif sur un trichobézoard.

## Patient et observation

**Figure 1:**
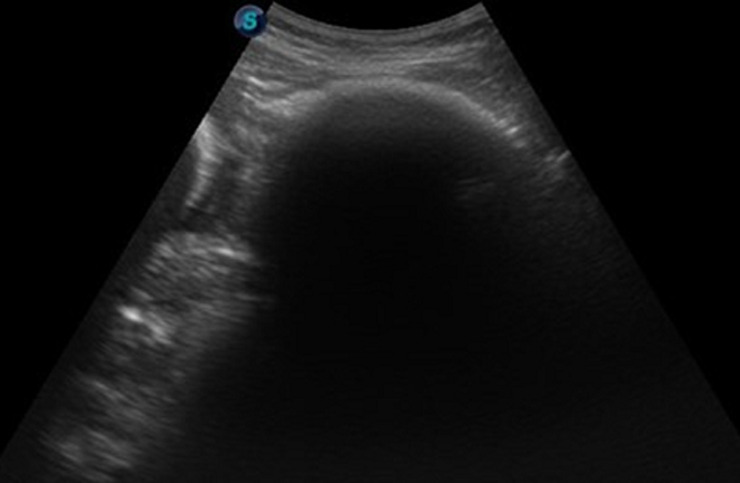
échographie abdominale: arc hyperéchogène épigastrique générant un cône d'ombre postérieur gênant l'exploration

Nous rapportons le cas d'une enfant âgée de 13 ans, de sexe féminin, suivie pour anémie sévère avec notion de trichotillomanie, admise au service de radiologie pour le bilan d'une douleur abdominale diffuse avec un maximum au niveau épigastrique, associée à un syndrome occlusif fait de vomissements alimentaires post-prandiaux précoces avec arrêt des matières et des gaz. A l'examen clinique, on retrouve une patiente consciente, apyrétique avec une plaque d'alopécie associée à une volumineuse masse épigastrique, ferme, indolore, mobile, non pulsatile. Une échographie abdominale a été réalisée (Figure 1), elle a mis en évidence un arc hyperéchogène épigastrique générant un cône d'ombre postérieur gênant l'exploration de la cavité abdominale.

**Figure 2:**
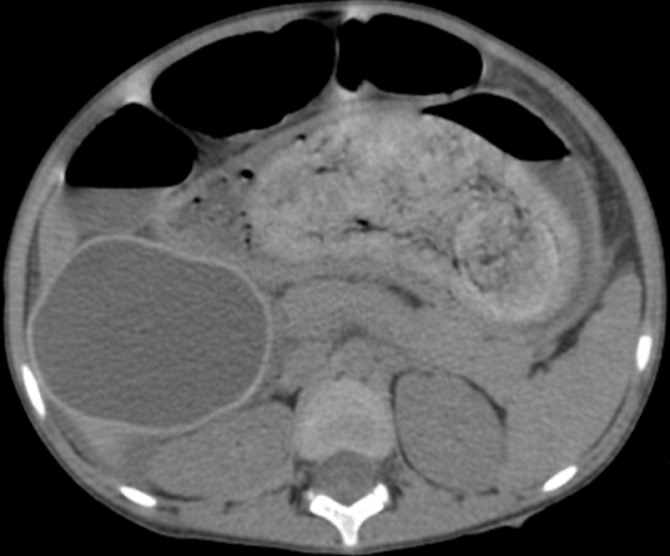
coupe axiale scanner abdomino-pelvien sans et après injection de PDC: trichobézoard à localisation gastrique avec extension duodénale associé à un KHF de type 1

**Figure 3:**
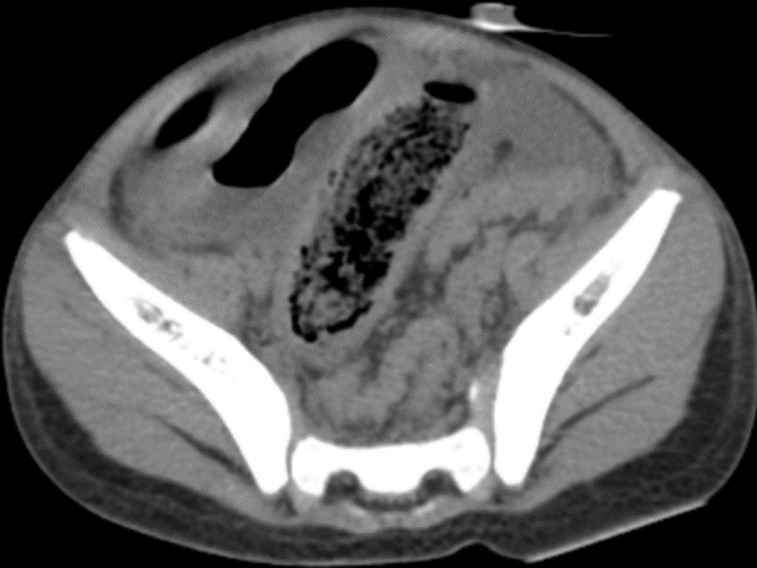
coupe axiale scanner abdomino-pelvien sans et après injection de PDC: localisation grêlique du trichobézoard: syndrome de Rapunzel

**Figure 4:**
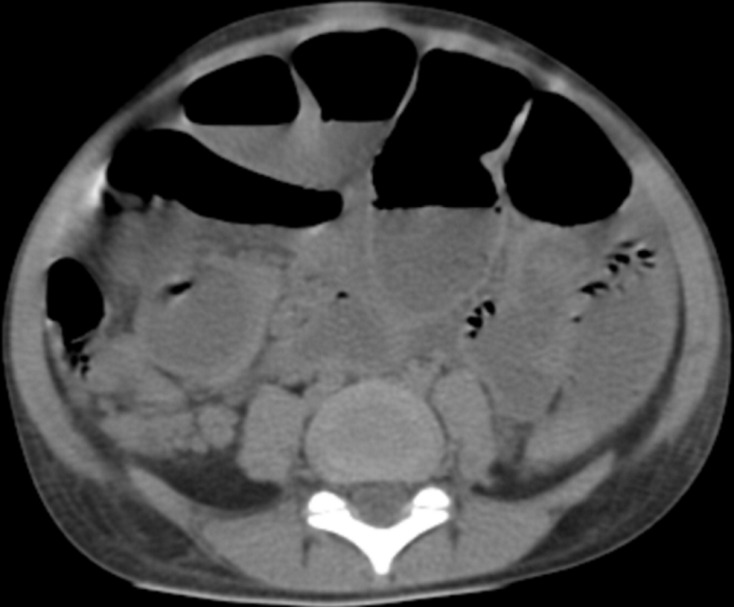
coupe axiale scanner abdomino-pelvien sans et après injection de PDC: occlusion mécanique grêlique en amont de l'obstacle

Nous avons complété le bilan par un scanner abdomino-pelvien avant et après injection de produit de contraste (PDC) (Figure 2), il a objectivé une distension gastrique et duodénale (D1 et D2) modérée, siège d'un matériel hétérodense, silhouetté par un liseré aérique périphérique avec des bulles d'air piégées. Ce matériel ne présentait pas d'attache pariétale et ne s'est pas réhaussé après injection de PDC. Il s'y associe une autre formation au niveau de la lumière grêlique distale de mêmes caractéristiques (Figure 3) responsable d'une occlusion grêlique avec un épanchement péritonéal de moyenne abondance mais sans signe de souffrance de la paroi digestive (Figure 4). On a noté également un kyste hydatique du foie (KHF) de type I au niveau des segments V et VI (Figure 2).

**Figure 5:**
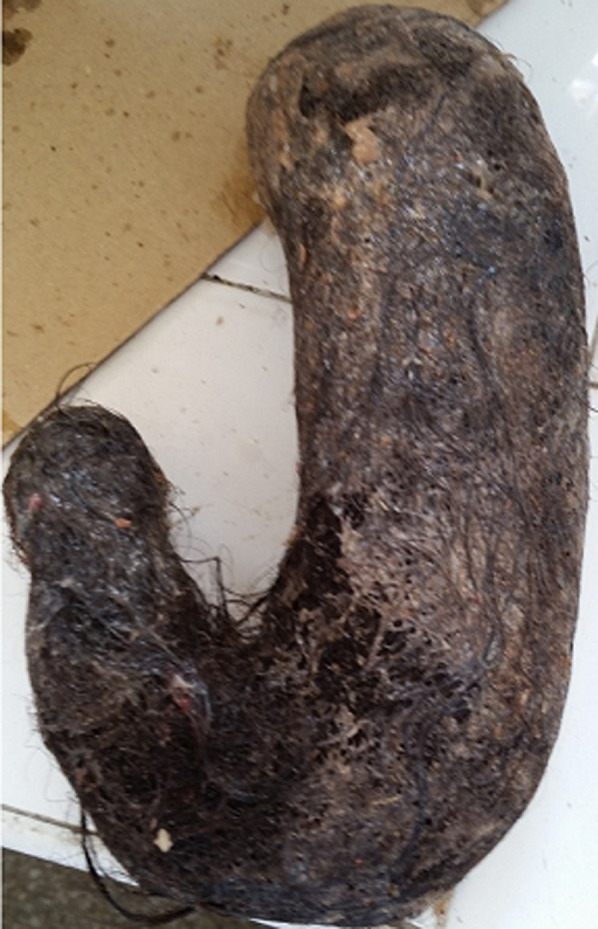
pièce anatomopathologique du trichobézoard

Le diagnostic d'occlusion intestinale mécanique sur trichobézoard intra gastrique avec localisation grêlique a été posé: réalisant un syndrome de Rapunzel. La patiente a été opérée ; elle a eu une gastrostomie longitudinale et une entérotomie avec extraction des bézoards gastrique et grêlique (Figure 5). Une ponction-aspiration-résection du dôme saillant du KHF a été aussi réalisée. Les suites post-opératoires étaient simples. La patiente a été adressée en pédopsychiatrie pour suivi et complément de prise en charge.

## Discussion

Les bézoards correspondent à la concrétion de substances ingérées variées non digestibles (phytobézoard, trichobézoard, lactobézoard, pharmacobézoard) dans le tractus gastro-intestinal. Le trichobézoard est le plus fréquent des bézoards. Le trichobézoard a été décrit pour la première fois en 1779 par Baudamant, il est fait de cheveux, poils ou fibres de tapis ou moquette de taille variable, formant une masse intra-gastrique pouvant avoir parfois des prolongements dans le duodénum, le jéjunum et même au-delà appelé «syndrome de Rapunzel» [1].

Le syndrome de Rapunzel a été rapporté par Vaughan en 1968 et tire son nom du conte des frères Grimm «Raiponce» écrit en 1812, qui rapporte l'histoire d'une princesse aux longs cheveux blonds emprisonnée dans une tour [2]. Dans 80% des cas, l'âge de survenue du Trichobézoard est inférieur à 30 ans avec un pic de fréquence entre 10 et 19 ans. La prédominance féminine est nette (90% des cas). Le trichobézoard se voit souvent chez des patients présentant des désordres psychologiques qui avalent leurs cheveux (trichophagie) après les avoir arrachés (trichotillomanie) mais en pédiatrie seulement 9% des enfants présentant un trichobézoard auraient de réels problèmes psychiatriques [3-5].

La symptomatologie clinique est variée et non spécifique. Longtemps asymptomatique, il peut se révéler par des douleurs abdominales, nausées, vomissements, éructations, haleines fétides, trouble du transit, amaigrissement, anorexie [5]. Le trichobézoard peut-être également découvert fortuitement lors d'un bilan d'anémie hypochrome microcytaire, d'hyperleucocytose ou d'hypoalbuminémie qui sont en rapport avec une malabsorption du fer et/ou un déficit en vitamine B12. Dans les cas extrêmes, il peut être diagnostiqué à la suite de complications graves dont les principales sont une hémorragie digestive haute sur ulcération, une péritonite par perforation digestive, une occlusion intestinale, une invagination intestinale aiguë, une pancréatite aigüe ou cholestase (obstruction de l'ampoule de Vater sur œdème réactionnel) [6, 7].

A l'examen clinique, on peut-dans certains cas-retrouver une plaque d'alopécie mais aussi dans la majorité des cas une masse épigastrique bien limitée, lisse, ferme, indolore, mobile, non pulsatile qui pose un problème de diagnostic différentiel en pédiatrie avec une tumeur du foie gauche, une splénomégalie, un neuroblastome ou une tumeur gastrique [8]. En cas de localisation gastrique ou intestinale proximale, la fibroscopie oeso-gastro-duodénale est l'examen de référence permettant de confirmer le diagnostic en objectivant des cheveux enchevêtrés ayant un aspect noir comme du goudron. Elle peut également avoir un intérêt thérapeutique en permettant l'extraction de bézoards gastriques de petite taille, mais elle n'est pas adaptée aux bézoards de grande taille. Cependant, elle ne permet pas d'identifier d'autres localisations au niveau intestinal distal qui peuvent être source de récidive sous forme d'une occlusion si elles ne sont pas traitées [7].

En imagerie, le diagnostic de trichobézoard est basé sur le trépied radiographie standard-échographie-scanner [9]. L'aspect typique du bézoard sur la radiographie standard abdominale-à savoir une masse intra-gastrique moulée par l'air-est retrouvé dans 18% des cas. Parfois, on peut visualiser un aspect fin des cheveux dans la lumière gastrique. La présence de niveaux hydro-aériques témoigne d'occlusion intestinale [9]. Le scanner a un double intérêt: diagnostique et pronostique. Il montre une masse intraluminale, ovoïde, hétérodense, sans attache à la paroi intestinale avec de minuscules bulles d'air intra lésionnelles dispersées. Il recherche également une dilatation des anses en amont, des signes de souffrance intestino-mésentériques ainsi que la localisation de ou des obstacles, ce qui a un grand intérêt dans la voie d'abord chirurgicale.

L'IRM n'est pas indiquée dans l'exploration de ce syndrome car la faible intensité de signal du bézoard peut être confondue avec l'air digestif [2]. Le traitement est endoscopique ou chirurgical en fonction de la taille et des différentes localisations du bézoard [10]. Le pronostic de cette affection est le plus souvent favorable néanmoins des complications peuvent survenir parfois létales notamment dans le cadre de localisations intestinales distales passées inaperçues ou de négligence du diagnostic et de la prise en charge psychologique [7].

## Conclusion

Le trichobézoard est important à connaître vu son aspect typique en imagerie permettant ainsi de guider la thérapeutique: chirurgicale ou endoscopique et d'orienter les patients vers une psychothérapie.
